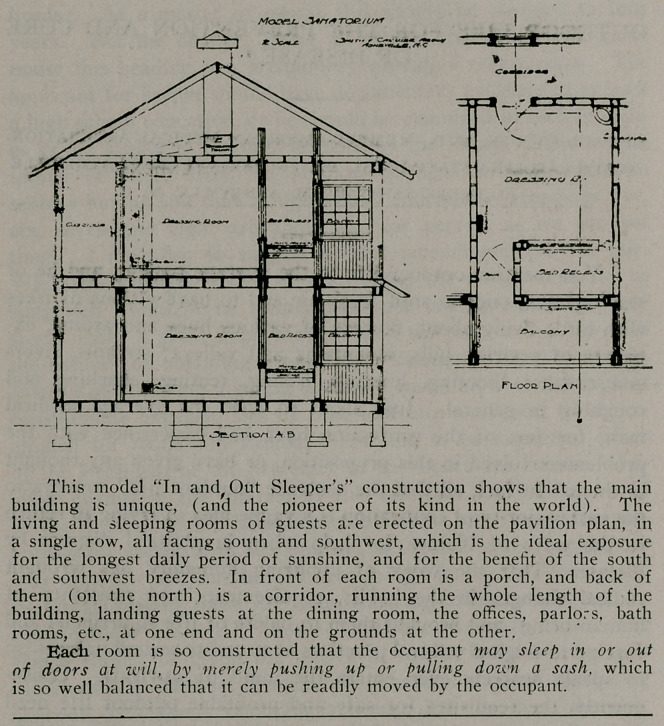# Outdoor Life for the Prevention and Cure of Disease

**Published:** 1908-07

**Authors:** Paul Paquin

**Affiliations:** Member American Medical Association, North Carolina State Local, etc., Superintendent Asheville-Biltmore Sanitarium, Asheville, N. C.


### OUTDOOR LIFE FOR THE PREVENTION AND CURE OF DISEASE.*

BY PAUL PAQUIN, M. D., MEMBER AMERICAN MEDICAL ASSOCIATION, NORTH CAROLINA STATE LOCAL, ETC., SUPERINTENDENT ASHEVILLE —BILTMORE SANITARIUM, ASHEVILLE, N. C.

You mention outdoor life to the average patient, and he or she begins at once to sniff fresh air and to have visions of trees with birds flying about, flowers of various hues and aroma, expanses of prairies, hills, mountains and valleys; streams, rivers and oceans; shooting, hunting, fishing, tenting, shacking and roughing in general. Just about so with the average medical man, for few of the profession have had experience with the problems involved in this proposition, or have given any thought to them. In fact, all that the sentence "outdoor life" conveys to the vast majority of civilization is a vague idea of trying to return to the ways of Nature for help when in physical distress by disease. That the work of civilizing humanity has wrought radical changes in the habits, requirements and resistance of human beings and brought them to habits of life so artificial and foreign to original natural practices as to create second natures, so to speak, scarcely ever enters the mind of anybody. Consequently, the requisites for safe and profitable outdoor life need to be discussed and studied if we would apply it in the prevention and cure of disease; and, as a preamble, I do not hesitate to say that reckless and haphazard outdoor life is no more justifiable than reckless and haphazard drugging. Both have had in the past, and have today graveyard consequences.

*Read before the North Carolina State Association, June 17, 1908.

Outdoor life goes back to primitive ages for its inspirations and ideals. The student of nature has discovered that certain maladies, particularly tuberculosis, are diseases of civilization. He has established that the wild man in the woods and jungles is seldom tubercular in his natural home in the open, and becomes involved by contact with civilized humanity. And so, the consumptives and their medical advisors prescribe outdoor life, which is well. But pause and consider. Don't push a sick man, woman or child out of doors in ignorance of individual conditions, circumstances and susceptibilities. As a rule the majority are not ready to cope instantly with and be benefitted by the influences of outdoor life in all its forces and moods.

Housing, clothing and inheritance have turned man from wild ruggedness into a hot house plant not always to be exposed without prior preparation to certain atmospheric and climatic conditions, with impunity. You should not expect, for instance, that a man who comes to you as many do, with an overcoat in the month of June, thick coat and vest underneath, a thick flannel shirt under these, one or more undershirts, then a chest protector as dense as cowhide, and finally the skin bound down by sticking plasters—you need not expect, I say, that such a man, stewed in the sweat and filth of his own body, can be suddenly laid out in the open air night and day without some sort of dangerous reaction. The best plan to catch a cold, and also for the pulmonary sufferer to develop a pulmonary congestion, is to sweat and then be exposed to a sudden process of cooling. This is only one way, however, to cause damage by outdoor life. There are many others, and so we need to understand them in order that we may not cause more harm than good in forcing patients to live out constantly.

As fundamental forces for beneficial outdoor life, we may point out above all things, the persistent supply of oxygen with the persistent dissipation of carbonic acid gas without rebreathing it, as compared with the more or less impoverished oxygen supplied in a room, and the constant re-breathing of the carbonic acid gas emanating from the lungs, and the breathing of house air laden with dusts and the dangerous germs of carpets, crannies and darkness, for, in the open air, in favorable localities at least, the germs that float about and which when inhaled might do harm if they had been protected within a building, are comparatively harmless after having been subjected to the influences of sunlight, rains and other atmospheric conditions. These basic factors are the essentials for the prevention and cure of disease by outdoor life. They are immensely valuable contributions to complete nutrition which is the very soul of health. Now, how are we to maintain safely, their good influence about a person, brought more or less out of close communion with Nature by ages of ancestral artificial existence and by individual artificialism and false methods of the present day civilization?

To begin with, we must educate each person, if found necessary, in a manner to restore as nearly as can be normal personal existence as to clothing and exposure to the various elements of the atmosphere. A person who overdresses and macerates his or her skin with perspiration, must first of all be gradually relieved of the incumbrance and made to live and to remain dry and to become capable of resisting the coolness of the breezes at least, not to say anything about accidental drafts and winds which all humans at times must be prepared to meet A person must also be taught that cold weather, rains, snows, etc., are not insalubrious—far less than heat perhaps—but that certain conditions affecting these or the persons subjected to them, are damaging; for instance, high winds, storms, direct drafts, sand or dust blowing moisture in low altitude, etc. Above all things, those persons who think that they protect themselves from cold by keeping wrapped up in shawls, coats, sweaters, blankets, etc., as suggested above, and who insist either of themselves or through persuasion, in living day and night in heavy underwear in order to keep off colds, should be taught and made to realize that this sort of so-called protection is a trap to catch colds and congestions and all the attendant catarrhs, and that underwear is primarily to keep the skin and necessary clothing far enough apart to maintain a free ventilation all around the body.

On the other hand, the question of sleeping in or out of drafts, of being close to or far above ground, of getting wet and chilled, or suddenly hot after being cold, of rising at night when sleeping out, are to be considered by doctors and patients, particularly those who preach tents and shacks without ever thinking of surroundings, arrangements and appointments thereof.

In lying down, night or day, one cannot afford to be in a direct draft that strikes only a part of the body. Be it ever so slight, it chills that part more or less, and, except perhaps when the draft hits the face alone, I assure you that usually a cold results. A breeze or light draft that is *not seriously chilling* in its temperature, and which covers and surrounds all the body alike, is not likely to cause a cold or congestion. The ways and wherefores of these facts cannot be discussed in this brief paper. Suffices it to sasert them to be a matter of experience.

It is evident then, that one who would live out night and day must be so situated as to be bathed constantly in fresh air with the minimum chances of being subjected to drafts. I need not say of course, that equal protection against chilling winds and rains and snows must also obtain. How can these desired results be secured? Simply by good common sense selection of a locality to live in, and proper arrangements to live out.

*First.*—Select a fairly high altitude as far from smoke and heavy dusts as possible.

*Second.*—If you choose a shack or tent, have its floors several feet above ground, and be *sure* that it is lavishly ventilated (without direct body drafts), for a shack or a tent of the usual kind is a fraud and a snare, and worse than an ordinary bedroom with open windows. For one thing, a canvas room is usually stuffy and hot, and then it is generally too close to the ground and absorbs moisture with rheumatic and congestive effects.

*Third.*—You might roost up a tree by arranging there some sort of hold-on-to-platform and overhead covering. This suggestion offers opportunities for profitable fresh air life undreamt of by the sick or the profession. It has been tried too, and found less wanting than most any other outing abode, although it appears absurd.

*Fourth.*—By far the most satisfactory arrangement is the specially devised outdoor living and sleeping rooms, called the "in and out sleeper," whereby one may lie day and night absolutely and completely in the open, surrounded by fresh air always, without any danger from drafts, rains, snows, winds, dusts, etc. This is the method presented last year to the section of hygiene of the American Medical Association. This system obtains today at the Asheville-Biltmore Sanitarium. No weather was ever found bad enough to do the least damge to guests in such quarters. Through the winter in dry and wet weather, everybody slept out. A special draft system at the foot of the bed draws the air past the body without touching it. keeping fresh air through and through in constant circulation, and then a foul air flue system carries the foul air of the rooms to an exit on top of the roof of the building.

In conclusion let me suggest that, after all, outdoor life is merely an aid to suitable nutrition, which is a process that involves not only the use of food and water, but the fullest possible assimilation of all the elements necessary to maintain the equilibrium of physical and mental forces in man, which we call health.

---

The thirty-fourth annual meeting of the Mississippi Valley Medical Association will be held in Louisville, Ky., October 13, 14, 15, 1908, under the presidency of Dr. Arthur R. Elliot, of

## Figures and Tables

**Figure f1:**